# Calcareous Bio-Concretions in the Northern Adriatic Sea: Habitat Types, Environmental Factors that Influence Habitat Distributions, and Predictive Modeling

**DOI:** 10.1371/journal.pone.0140931

**Published:** 2015-11-11

**Authors:** Annalisa Falace, Sara Kaleb, Daniele Curiel, Chiara Miotti, Giovanni Galli, Stefano Querin, Enric Ballesteros, Cosimo Solidoro, Vinko Bandelj

**Affiliations:** 1 Department of Life Sciences, University of Trieste, Trieste, Italia; 2 SELC Society Marghera, Venezia, Italia; 3 National Institute of Oceanography and Experimental Geophysics-OGS, Trieste, Italia; 4 Centre d’Estudis Avançats de Blanes-CSIC, Girona, España; Università di Genova, ITALY

## Abstract

Habitat classifications provide guidelines for mapping and comparing marine resources across geographic regions. Calcareous bio-concretions and their associated biota have not been exhaustively categorized. Furthermore, for management and conservation purposes, species and habitat mapping is critical. Recently, several developments have occurred in the field of predictive habitat modeling, and multiple methods are available. In this study, we defined the habitats constituting northern Adriatic biogenic reefs and created a predictive habitat distribution model. We used an updated dataset of the epibenthic assemblages to define the habitats, which we verified using the fuzzy k-means (FKM) clustering method. Redundancy analysis was employed to model the relationships between the environmental descriptors and the FKM membership grades. Predictive modelling was carried out to map habitats across the basin. Habitat A (opportunistic macroalgae, encrusting Porifera, bioeroders) characterizes reefs closest to the coastline, which are affected by coastal currents and river inputs. Habitat B is distinguished by massive Porifera, erect Tunicata, and non-calcareous encrusting algae (*Peyssonnelia* spp.). Habitat C (non-articulated coralline, *Polycitor adriaticus*) is predicted in deeper areas. The onshore-offshore gradient explains the variability of the assemblages because of the influence of coastal freshwater, which is the main driver of nutrient dynamics. This model supports the interpretation of Habitat A and C as the extremes of a gradient that characterizes the epibenthic assemblages, while Habitat B demonstrates intermediate characteristics. Areas of transition are a natural feature of the marine environment and may include a mixture of habitats and species. The habitats proposed are easy to identify in the field, are related to different environmental features, and may be suitable for application in studies focused on other geographic areas. The habitat model outputs provide insight into the environmental drivers that control the distribution of the habitat and can be used to guide future research efforts and cost-effective management and conservation plans.

## Introduction

Coralligenous outcrops are among the most diverse and representative Mediterranean benthic ecosystems, and they are produced by the interplay between calcareous organism building processes and physical and biological erosional processes [1]. Several types of coralligenous morphologies have been identified in the literature [1–7]. The main recognized morphologies are reef banks, which are flat structures (ranging from 0.5 to 4 m in height) built over more or less horizontal substrates, and coralligenous rims, which are structures that grow on vertical cliffs and are generally located in shallower waters [1,7–9].

Most scientists consider coralligenous outcrops to be seascapes or community mosaics rather than a single community. These biogenic structures are complex and contain areas dominated by algae, suspension feeders, borers, or even soft-bottom fauna living in the sediment deposited in cavities and holes [10]. Certain dominant species that characterize the calcareous bio-concretions are long-lived engineering species, which makes this habitat extremely vulnerable to disturbances [1,10–12].

Because of their extent, biodiversity, and implications for fisheries and carbon regulation, calcareous biogenic habitats are considered priority habitats at the European and regional levels [10,13,14].

Marine habitat classifications are performed to provide standard nomenclature and guidelines for describing, mapping, and comparing marine environments and associated assemblages across geographic regions [15]. Moreover, habitat classifications assist in the management of marine resources and the quantification of ecosystem processes and services at different temporal and spatial scales. Finally, habitats can be used as a surrogate for biodiversity, and they provide guidance for monitoring programs [16]. For example, the identification of thresholds between the ecological statuses of priority habitats in the European Marine Strategy Framework Directive (MSFD, 2008/56/EC) of “Good” and “Not Good” is based on “Habitat Distribution,” “Habitat Extent”, and “Habitat Condition.”

The geomorphological features of coralligenous build-ups and their associated biota have not been exhaustively categorized. In particular, coralligenous build-ups that occur in areas where boulders are associated with sand and mud, such as in the northern Adriatic Sea to the Apulia region, should be considered a specific type [17]. According to the European Habitats Directive (92/43/EEC), the marine rocky outcrop classification is included in the Annex I habitat types as “1170-Reefs” (36). In the context of the Barcelona Convention (UNEP/OCA/ MED WG149/5 Rev. 1, 2006), which is an elaboration of the CORINE biotopes nomenclature [18], coralligenous biocoenosis (IV.3.1) is included within the circalittoral hard beds and rocks categories and contains 15 different facies [10]. Finally, according to the MSFD, coralligenous biocoenoses fall into the categories “*Facies and associations of coralligenous biocoenosis (III*.*6*.*1*.*35)*” and “*Shallow sublittoral rock and biogenic reef*”. However, these bulk categories are not appropriate for management purposes because they each encompass a large range of biogenic natural habitats that can differ significantly in their ecological and conservation features [19]. Europe generally employs the European Nature Information System (EUNIS) habitat classification scheme ([20]; http://eunis.eea.europa.eu); however, the development of the marine EUNIS classification is primarily based on Atlantic ecosystems, whereas Mediterranean ecosystems are roughly incorporated into the EUNIS list using habitats from the Barcelona Convention. Thus, coralligenous habitats are currently classified as “A4.26: *Mediterranean coralligenous communities moderately exposed to hydrodynamic action*” and “A4.32: *Mediterranean coralligenous communities sheltered from hydrodynamic action”* in the EUNIS system.

Despite their ecological, aesthetic, and economic value, complete and up-to-date baseline information on coralligenous outcrops is not available [11], and most of the current information is derived from the western Mediterranean [14], where coralligenous outcrops are unlikely to occur in sedimentary zones, enclosed estuarine environments, and sandy areas with low salinities, such as river mouths [14]. However, hundreds of calcareous bio-concretions are scattered on the muddy-detritic bottom of the northern Adriatic Sea. These biogenic outcrops are considered to have a significant degree of similarity with coralligenous outcrops [21] [22] [23], although their composition and overall structure show striking differences [23], and according to the EUNIS classification, they should be classified as a different habitat.

The increasing awareness of the importance and fragility of these habitats has led to global efforts to conserve these ecosystems according to several legally binding or voluntary international initiatives. For environmental research, resource management, and conservation planning, mapping is critical, although it is not an easy task in marine habitats that might be distributed over hundreds of square kilometers. In recent decades, many developments have occurred in the field of species and habitat distribution modeling, and multiple methods are now available [24,25]. The construction of a geographical distribution model requires observations of species/habitat occurrences and environmental variables that are considered to influence habitat suitability [26]. The quantification of such species–environment relationships represents the foundation used to predict the likelihood of a species occurring at a given location [25].

Currently, predictive habitat modeling is performed at regional or global scales and appears to be a cost-effective method of identifying the location of vulnerable marine habitats, such as coralligenous reefs, although this modeling does not provide habitat maps. Predictive habitat modeling provides insight into the environmental drivers that control the distribution of vulnerable marine habitats and can be used to guide research efforts [14,27,28].

In this study, we intend to provide (1) a definition of the different habitats constituting northern Adriatic biogenic reefs, (2) an assessment of the main physical and environmental variables accounting for their distribution and (3) a predictive habitat map to indicate the occurrence of biogenic reef habitats in the northern Adriatic Sea.

## Material and Methods

### Study area

The northern Adriatic Sea is the most dynamic sub-basin of the Mediterranean Sea [29,30], and it is characterized by strong river runoff and wide seasonal and interannual variability in temperature and salinity. The Adriatic Sea is surrounded by mainland areas that exhibit sharp contrasts in tectonism, topography, climate, and fluvial inputs. Northwestern Adriatic shores are sedimentary and contain a continuous line of coastal lagoons. The water density gradient between the northern and the southern Adriatic Sea is the most important factor that triggers the movement of water in a primarily counterclockwise current that flows down to the Otranto Strait and into the Mediterranean Sea [31]. River discharges show a remarkable seasonality, with the highest flow rates usually occurring in late spring and autumn. The concentration of inorganic nutrients is highly variable and is mainly related to river inputs [32].

From the Gulf of Trieste to the Po River delta, biogenic outcrops, locally known as “*tegnùe*” or “*trezze”*, are scattered on the soft bottom, and they were first identified as beachrocks [33–36]. Recent studies have related their genesis to seeping methane, cementation, and lithification processes [37–41]. These rocky outcrops are “calcareous bio-concretions” derived from the building action of calcareous organisms on hard substrata of diverse geological origins. The origins of the complex primary substrata consist of a carbonatic conglomeration of sandy sediments mixed with shells and other exoskeletons. The buildup process may be accelerated by the seepage of methane through the sediments and by subtidal freshwater streams [40]. The calcareous bio-concretions display a broad range of geomorphologies and extend from a few to several thousands of square meters. The offshore bio-concretions situated in front of the Venice Lagoon are sloped and stretch parallel to the coast. Several outcrops show large horizontal surfaces, whereas others are composed of scattered conglomerates of small rocks. The surrounding sea floor is mainly detritic because it accumulates skeletons of species growing either in the sediment or in the neighboring outcrops.

### Habitat typology

We used an updated spatial dataset based on information provided by peer-reviewed articles, regional, national, and international reports and by recently unpublished data obtained by the authors to produce an overview of the epibenthic assemblages associated with the northern Adriatic calcareous bio-concretions. Data on macroalgal assemblages were obtained from studies performed over an approximately 30-year period [22,23,42–47] as well as from recent studies. Data on benthic invertebrates were obtained from peer-reviewed articles [21,22,48–52] and national unpublished reports [53–55].

**Fig 1:**
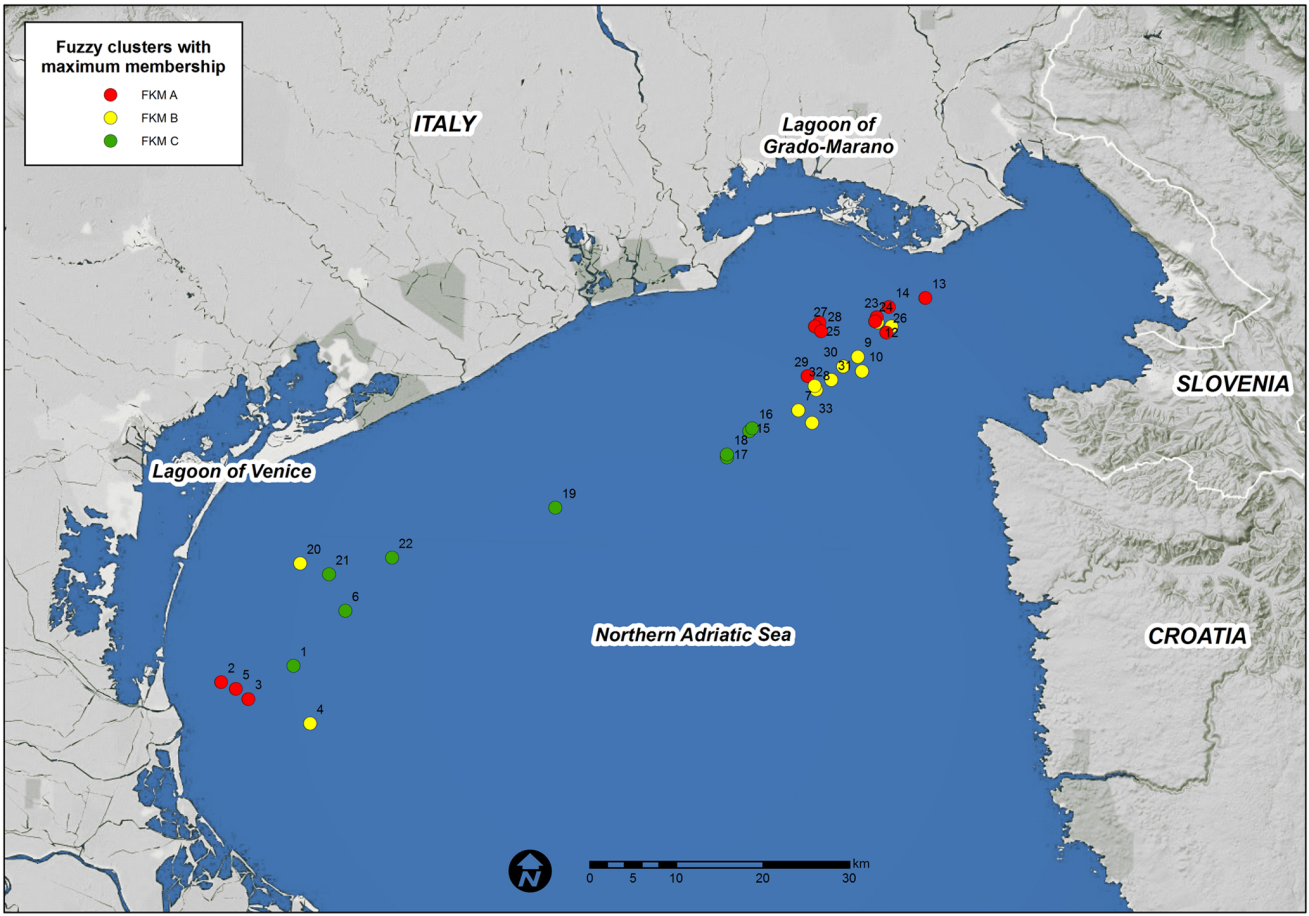
Occurrences of the 3 habitat typology outcrops across the northern Adriatic Sea (original copyright 2015).

Habitat typology was established by expert judgment based on knowledge of the assemblages and the updated dataset. This typology was then verified on a large scale using 33 outcrops for which comparable data were available (Fig 1).

### Fuzzy clustering methods

To evaluate the habitat typology produced through “a priori expert judgment,” we used the fuzzy k-means (FKM) clustering method [56] and performed the clustering with the parameter of fuzziness set to 2 and the number of random initializations set to 1000. All FKM calculations were performed using the fclust package for R [57].

### Environmental database

Data on water temperature, salinity, dissolved oxygen and chlorophyll a, and ammonium, nitrate and phosphate concentrations were extracted from the dataset of vertical seawater profiles collected by i) Solidoro et al. [58] from 1986–2006; ii) the Regional Water Authority (ARPA Veneto, 1985–2004) in monthly or biweekly measurements performed along 20 transects orthogonal to the Veneto coast and extending offshore up to 5 nautical miles; and iii) the Regional Water Authority (ARPA-FVG, 2009–2012) in monthly measurements performed at 21 monitoring stations along the Friuli-Venezia Giulia coastline (Table 1). The surface (shallowest record) and bottom (deepest record) values of all variables were extracted for winter (January, February, and March), spring (April, May, and June), summer (July, August, and September) and autumn (October, November, and December).

**Table 1:** Environmental descriptors, measurement units, and data sources. All of the variables except depth were extracted at the surface and the bottom.

ACRONYM	VARIABLE	UNITS OF MEASURE	Data source
TEMP	Water temperature (surface and bottom, median and range)	°C	Solidoro et al. (2009); ARPA Veneto; ARPA FVG
SAL	Salinity (surface and bottom, median and range)		Solidoro et al. (2009); ARPA Veneto; ARPA FVG;
DOX	Dissolved oxygen (surface and bottom, median and range)	mL L^-1^	Solidoro et al. (2009); ARPA Veneto
AMON	Ammonium concentration (surface and bottom, median and range)	μmol L^-1^	Solidoro et al. (2009); ARPA Veneto; ARPA FVG
NTRA	Nitrate concentration (surface and bottom, median and range)	μmol L^-1^	Solidoro et al. (2009); ARPA Veneto; ARPA FVG
PHOS	Phosphate concentration (surface and bottom, median and range)	μmol L^-1^	Solidoro et al. (2009); ARPA Veneto; ARPA FVG
CPHL	Chlorophyll a (surface and bottom, median and range)	μg L^-1^	Solidoro et al. (2009); ARPA Veneto; ARPA FVG
Vmean	Mean velocity (surface and bottom)	m s-1	Ocean circulation model
Vmax	Max velocity (surface and bottom)	m s-1	Ocean circulation model
Depth	Bottom depth	m	GEBCO 30 arc-second grid http://www.gebco.net/

A minimum depth of 5 m was imposed for the bottom values. We calculated the median seasonal values of each parameter on a 2.5 x 2.5 km grid, and we calculated a yearly median only if data were present for all 4 seasons to prevent biases caused by different sampling efforts in different seasons. Because the data were spatially sparse and a number of grid cells were left empty, we extrapolated information to grid cells without data by means of the moving window method [58]. For each cell, the median for at least 10 data points within the surrounding cells in a search radius of 20 km was calculated to determine the missing temperature, salinity, and chlorophyll a values. For the remaining variables, we used a search radius of 30 km and at least 6 data points. The same procedures were applied to derive ranges of variation between the 95^th^ and 5^th^ percentiles of distribution for each parameter at the surface and the bottom. We used the 95^th^ and 5^th^ percentiles instead of the absolute maximum and minimum values, respectively, to prevent occasional extreme data from biasing the range calculations. The gridded results of the median and value ranges for each surface and bottom variable were exported and geo-referenced as geographic information system (GIS) raster layers.

Hydrodynamic data were extracted from a high-resolution numerical model of the northern Adriatic Sea. The simulation was performed by customizing the MITgcm (Massachusetts Institute of Technology general circulation model), which is a three-dimensional, finite-volume general circulation model. The numerical experiment presents a higher resolution (4-fold higher, which is ~750 m in the horizontal direction) version of the simulation described in Querin et al. [59], and it is focused on the northern Adriatic Sea for the year 2008. The computational grid is composed of 30 vertical levels. The model neglects tides and short gravity waves (wind waves). For the bottom velocities, we sampled the first grid elements above the deepest cells to produce a fully developed velocity field and avoid boundary layer effects at the bottom. The velocities were averaged over a 2.5 x 2.5 km grid and then geo-referenced as GIS raster layers.

For the bathymetry, we downloaded the GEBCO 30 arc-second grid from the General Bathymetric Chart of the Ocean (GEBCO 2014. Database: GEBCO_2014 Grid version 20150318; http://www.gebco.net/) and extracted data at the coordinates of the outcrops as well as data on the 2.5 x 2.5 km grid to ensure consistency among the explanatory variables.

All of the GIS computations were performed using QGIS [60].

### Direct gradient analysis method

A redundancy analysis (RDA) [61,62] was used to model the relationship between the environmental descriptors and the FKM membership grades [63]. The biotic data table was transformed using the Hellinger transformation [64] prior to performing the RDA to avoid the species abundance paradox [65].

The number of environmental predictors was the same as the number of samples; therefore, an RDA with all of the environmental variables would not be constrained. Furthermore, it has been shown that explained variance continues to increase when including variables, even if they are random or insignificant [66,67]. To reduce the number of explanatory variables while still preserving their explanatory power, we chose a two-step procedure and divided the explanatory variables into 3 subsets: a subset including the median and value ranges for 7 water quality parameters at the surface; a subset including the median and value ranges for 7 water quality parameters at the bottom subset; and a hydrodynamic subset including values for 4 variables. For each of these subsets, an RDA was performed, the axes were tested for significance, and the significant explanatory variables were selected by forward selection using a double stopping criterion [68]. The significant explanatory variables of each subset were then used along with the depth values as the explanatory variables of the final RDA model. Variation partitioning [69] was applied to the 3 groups of variables and the depth values in the final RDA model to study their mutual relationships.

To predict the fuzzy cluster membership grades over a grid covering the Italian sector of the northern Adriatic, we applied canonical coefficients from the final RDA model to the values of the selected environmental variables in each of the 2.5 x 2.5 km bins. The results were projected in GIS as geo-referenced raster maps.

All of the analyses were performed using the vegan [70], ade4 [71] and packfor [72] packages for R.

## Results and Discussion

### Habitat classification

#### Biodiversity

Most of the studies conducted on the epibenthic assemblages of the northern Adriatic bio-concretions are qualitative. Only the most recent research ([22,23,51]; unpublished data) has reported quantitative data, although these studies are generally restricted to small or medium spatial scales or consider the flora and fauna separately. A total of 573 taxa have been reported, which includes a relatively high number of macroalgae (191 taxa) (S1 Checklist) considering the biogeographical context and the dispersal of outcrops on muddy sandy bottoms far from coastal sources of spores and propagules. More shallow bio-concretions are mainly characterized by taxa that are widespread in nearby lagoons [73–78] and the Gulf of Trieste shoreline [79], and they include turf-forming or laminar taxa. All of the calcareous species of macroalgae, which are acknowledged as the most important coralligenous bioconstructors [80–82], have been reported, even if most have low coverage. The highest coverage of bioconstructors, particularly *Lithophyllum incrustans* and *Mesophyllum* spp., is found on the outcrops located at a depth of 23–25 m and at a distance ≥10 km from the coast. However, a number of common coralligenous taxa [10] are found in low amounts or at extremely rare frequencies (i.e., *Palmophyllum crassum*, *Flabellia petiolata*, *Halimeda tuna*). The most numerous of the 382 animal taxa are Mollusca (107 taxa), Polychaeta (92 taxa), Porifera (59 taxa), and Crustacea (50 taxa) (S2 Checklist). Most of these epibenthic invertebrates are filter feeders. The high number of Porifera appears to be a common feature of eastern Mediterranean coralligenous assemblages, which is most likely because of the absence of alcyonarians and gorgonians [83]. The “large animal builders” (*sensu*, [82]) reported here include the Serpulidae *Serpula vermicularis* and *Serpula concharum*, the Vermetidae *Thylacodes arenarius*, and the Anthozoa *Leptopsammia pruvoti*, *Caryophyllia inornata*, and *Caryophyllia smithii*. *Cladocora caespitosa* is rare on Italian outcrops, whereas it is an important builder in Slovenia [84]. On the Veneto outcrops, the fossil record testifies to the historical relevance of this bioconstructor [46]. The most common animals with “reduced builder activity” (*sensu*, [82]) are the Serpulidae *Hydroides* spp. and the Verrucidae *Verruca stroemia*. Finally, the “agglomerative builders” (*sensu*, [82]) include the Anthozoa *Epizoanthus arenaceus* and the Demospongia *Geodia cydonium*. Another characteristic feature of these northern Adriatic outcrops is the absence of large Bryozoa (i.e., *Margaretta cereoides*, *Cellaria salicornioides*, *Pentapora fascialis*, and *Reteporella grimaldii*), which are abundant in Mediterranean coralligenous environments. In the bioconstruction buildup an important counterpart to the biological carbonate deposition is the bioeroders activity [85]. A total of 11 bioeroders were found, which include 4 Porifera, 1 Sipuncula, 4 Bivalvia, and 2 Polychaeta (S2 Checklist). *Cliona viridis* and *Cliona celata* are the more common taxa, whereas *Cliona rhodensis* and *Cliona thoosina* were only found by Ponti *et al*. [22]. Microborers (i.e., fungi and cyanobacteria) have not been considered, whereas among the macroborers, the most frequent were the Mollusca *Hiatella arctica*, *Rocellaria dubia*, *Lithophaga lithophaga*, and *Petricola lithophaga*.

The most frequent and widespread taxa found on the northern Adriatic calcareous bio-concretions are reported in Table 2.

**Table 2:** Common and abundant taxa on northern Adriatic calcareous bio-concretions.

**Macroalgae**	*Aglaothamnion* spp.		*Suberites domuncula*
	*Cladophora spp*.		*Tedania (Tedania) anhelans*
	*Cryptonemia lomation*		*Tethya aurantium*
	*Dasya* spp.	**Anthozoa**	*Cereus pedunculatus*
	*Dictyota dichotoma*		*Cerianthus membranaceus*
	*Gracilariopsis longissima*		*Cornularia cornucopiae*
	*Halymenia floresii*		*Epizoanthus* spp.
	*Halopteris filicina*	**Mollusca**	*Arca noae*
	*Lithophyllum* spp.		*Bolma rugosa*
	*Lithothamnion* spp.		*Calliostoma zizyphinum*
	*Mesophyllum macroblastum*		*Haliotis tuberculata*
	*Nitophyllum punctatum*		*Hiatella arctica*
	*Peyssonnelia* spp.		*Ostrea edulis*
	*Pseudochlorodesmis furcellata*		*Rocellaria dubia*
	*Rhodophyllis divaricata*	**Crustacea**	*Dromia personata*
	*Rhodymenia ardissonei*		*Homarus gammarus*
	*Scinaia complanata*	**Echinodermata**	*Holothuria (Holothuria) tubulosa*
	*Taonia atomaria*		*Ocnus planci*
	*Zanardinia typus*		*Ophiothrix fragilis*
**Porifera**	*Antho (Antho) inconstans*		*Sphaerechinus granularis*
	*Aplysina aerophoba*	**Tunicata**	*Aplidium* spp.
	*Axinella* spp.		*Cystodytes dellechiajei*
	*Chondrosia reniformis*		*Microcosmus vulgaris*
	*Cliona viridis*		*Phallusia* spp.
	*Dictyonella incisa*		*Polycitor adriaticus*
	*Dysidea* spp.	**Polychaeta**	*Sabella spallanzanii*
	*Geodia cydonium*		*Serpula* spp.
	*Ircinia variabilis*		*Spirobranchus triqueter*
	*Sarcotragus spinosulus*		

#### Habitat types

**Fig 2:**
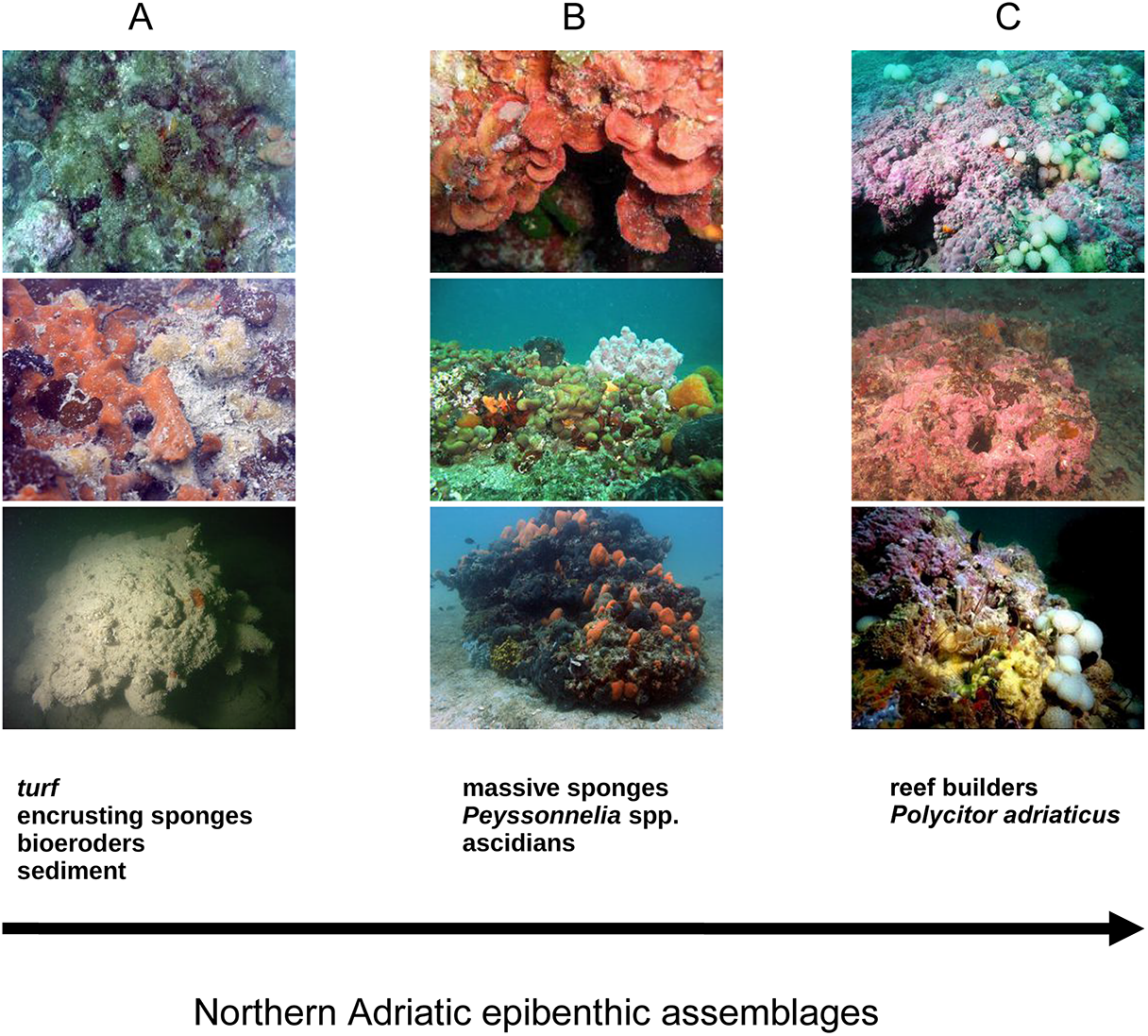
Dominant epibenthic assemblages of calcareous bio-concretions (Habitat A, Habitat B, and Habitat C) (original copyright 2015).

According to expert judgment, 3 dominant epibenthic assemblages have been distinguished (Fig 2).

The first group of reefs (Habitat A) is distinguished by opportunistic and tolerant macroalgal species that are resistant to mud and organic matter (i.e., turf-forming algae such as *Cladophora* sp., *Antithamnion* sp., and *Pseudochlorodesmis furcellata*); encrusting Porifera [i.e., *Antho (Antho) inconstans*, *Dictyonella incisa* and *Mycale (Mycale) massa*]; and bioeroders (i.e., *Cliona* spp. and *Rocellaria dubia*). A second group of reefs (Habitat B) is dominated by massive Porifera (i.e., *Chondrosia reniformis*, *Tedania anhelans*, and *Ircinia variabilis*); erect Tunicata (*Aplidium conicum* and *Aplidium tabarquensis*); and non-calcareous encrusting algae (*Peyssonnelia* spp.). The third group of reefs (Habitat C) is located in deep offshore waters and is dominated by non-articulated calcareous macroalgae and, to a lesser extent, by the tunicate *Polycitor adriaticus*.

### Fuzzy clustering

A comparison between the FKM results and the reef types based on expert knowledge is consistent (Table 3). Only 5 sites (ChioL2, Lastre, Corvine, Nordalti, and TR2-Pinnacoli) are assigned to a different type by the FKM cluster with the highest fuzzy membership. In all these cases, the expert type assignation is more “conservative” (Fig 2) compared with that of the FKM, i.e., the sites that were assigned by expert knowledge fell in the category immediately below the maximum membership category assigned by the FKM. Furthermore, two of these mismatches occur for sites that the FKM assigned high levels of fuzziness (ChioL2 and Nordalti). Thus, the three FKM clusters have been renamed according to the expert typology. High fuzziness levels, i.e., no membership >0.50, is observed in one-third of the studied sites. Among the remaining sites, 8 have FKM_A>0.50, 7 have FKM_B>0.50, and 8 have FKM_C>0.50. The FKM_B cluster shows the most restricted membership range (min 0.14 –max 0.60); however, both the FKM_A (0.07–0.70) and FKM_C (0.11–0.78) clusters clearly prevail at certain sites, while they show low membership values at other sites. These results supports the interpretation of the FKM_A and FKM_C clusters as the extremes of a gradient that characterizes the epibenthic assemblages on the outcrops. There is also a difference in the mean depth of the reefs of each cluster; the FKM_A and FKM_B clusters are found in shallower waters (17.6 m and 18.0 m, respectively), whereas the FKM_C reefs are found in deeper areas (22.6 m).

**Table 3:** Results of the FKM membership grades (FKM_A, FKM_B, and FKM_C) and habitat typology (Typ) to which the outcrop has been assigned based on expert knowledge (Habitats A, B, and C). The mismatches between the expert typology and the FKM results are in bold.

STATION	Typ	FKM_A	FKM_B	FKM_C
ChioL1	C	0.13	0.39	0.48
ChioS1	A	0.61	0.25	0.14
ChioS3	A	0.49	0.30	0.21
ChioL2	**B**	0.24	0.32	0.44
ChioS2	A	0.41	0.35	0.24
ChioL3	C	0.20	0.33	0.46
TR12-Nicola	B	0.19	0.49	0.32
TR13	B	0.19	0.54	0.28
TR14-Misto	B	0.19	0.52	0.29
TR3-Spari	B	0.23	0.49	0.29
TR4	B	0.30	0.47	0.22
SanPietro	B	0.22	0.60	0.17
Menegh	A	0.70	0.19	0.11
Meneghel	A	0.70	0.19	0.11
Strucolo	C	0.10	0.21	0.69
Gubana	C	0.08	0.15	0.77
Colomba	C	0.07	0.14	0.78
Colomba2	C	0.11	0.20	0.69
Cerniotta	C	0.09	0.17	0.74
Lastre	**B**	0.18	0.32	0.51
Pivetta	C	0.16	0.29	0.55
Tartaruga	C	0.15	0.26	0.59
Amerigo	A	0.54	0.32	0.14
Corvine	**A**	0.37	0.50	0.13
NordAlti	**A**	0.40	0.44	0.16
Palo Largo	A	0.66	0.22	0.12
TR2-Pinnacoli	**A**	0.27	0.51	0.22
Salient	A	0.63	0.26	0.11
Saratoga	A	0.52	0.35	0.13
Dorsale	B	0.37	0.42	0.21
Aldebaran	B	0.25	0.55	0.20
La Longa	B	0.34	0.44	0.22
Bardelli	B	0.16	0.51	0.32

### Direct gradient analysis according to the RDA

The surface chemical-physical model constructed with 14 variables (the median and value ranges for the surface TEMP, SAL, DOX, NTRA, AMON, PHOS, DOX, and CPHL) had an adjusted R^2^ [68,86] of 0.78 (0.63 on the first axis, 0.15 on the second axis, both p<0.001, 999 permutations). Many of the variables were not statistically significant because they presented high collinearity. The forward selection only retained four variables (PHOS, SAL, CPHL, and value range for CPHL) and had an adjusted R^2^ = 0.67 and first axis R^2^ = 0.60 (both axes p<0.01, 999 permutations) (S1 Fig).

The bottom chemical-physical RDA of 14 variables (the median and value ranges for the bottom TEMP, SAL, DOX, NTRA, AMON, PHOS, DOX, and CPHL) had an adjusted R^2^ of 0.58. The majority of the variance was explained by the first axis (0.49), although both axes were significant (p<0.001, 999 permutations). The forward selection only retained two variables (value ranges for TEMP and PHOS). The reduced model explained 0.58 of the variance on the first axis and 0.05 of the variance on the second axis (both axes p<0.05, 999 permutations) (S2 Fig).

The hydrodynamic model was built with all 4 hydrodynamic variables (Vmean and Vmax of the surface and bottom). The adjusted R^2^ was 0.23 on the only significant axis (p<0.001, 999 permutations). The forward selection retained only the two surface velocities, and the adjusted R^2^ was 0.26 on the only significant axis (p<0.001, 999 permutations) (S3 Fig).

**Fig 3:**
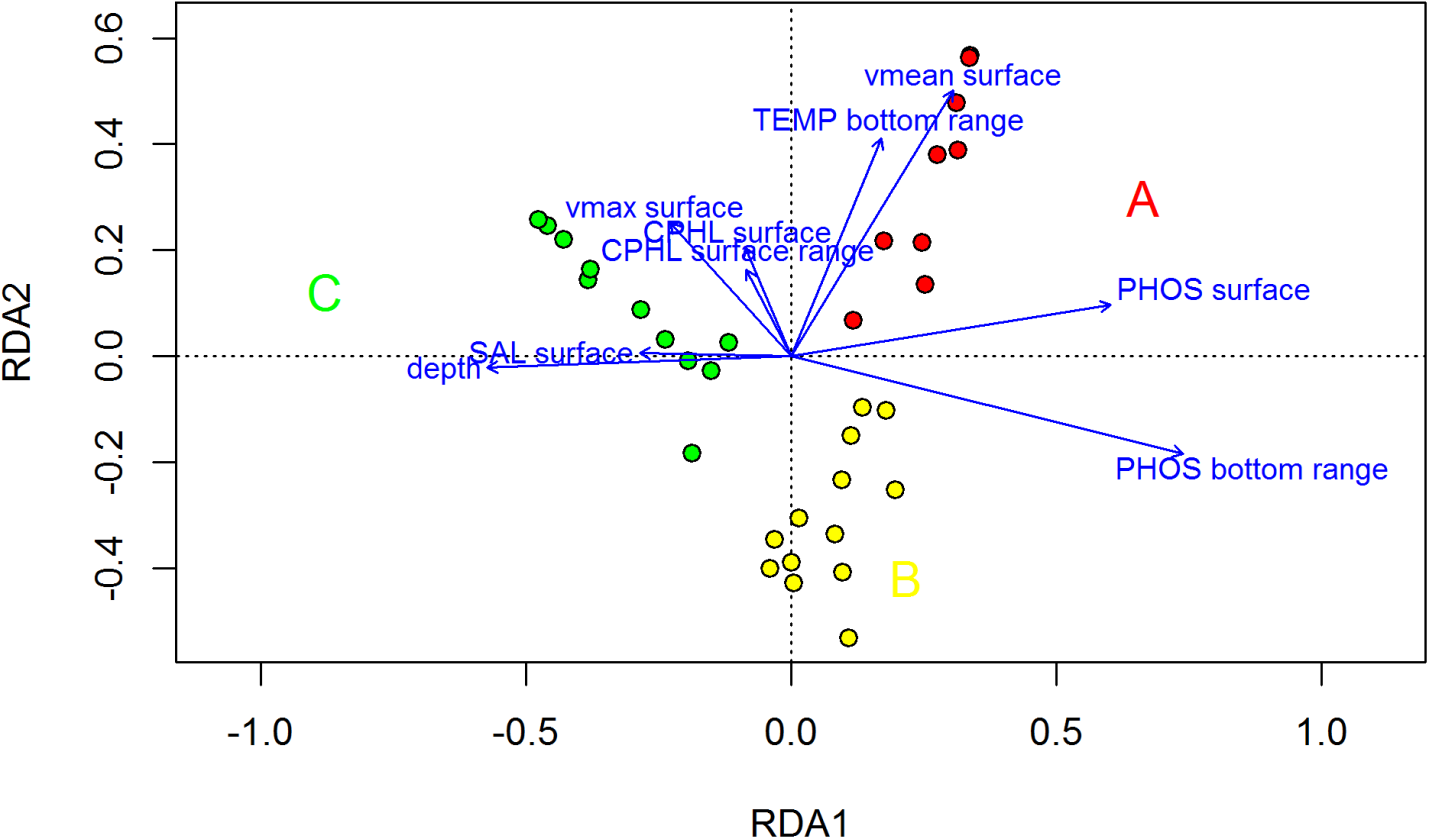
Final RDA model. Entire model adjusted to R^2^ = 0.79, first axis adjusted R^2^ = 0.64, second axis adjusted R^2^ = 0.14. Both axes are significant at p<0.001 after 999 permutations **(original copyright 2015)**.

The final RDA was built using the selected variables of the three RDA subsets: the median surface PHOS, SAL, and CPHL; value ranges for the surface CPHL; and value ranges for the bottom TEMP and PHOS, surface Vmean and Vmax, and depth. The entire model had an adjusted R^2^ of 0.79; 0.64 of the variance was explained by the first axis, and 0.14 of the variance was explained by the second axis, with both of these values highly significant (p<0.001, 999 permutations). This result was obtained using only 9 variables out of the initial 33. A further forward selection retained only 3 variables (range of phosphate at bottom, mean surface velocity, and surface phosphate), with an adjusted R^2^ of 0.74 on the two significant axes (p<0.001, 999 permutations). In the following, we discuss the final model with 9 partially redundant variables because many of them are of great ecological importance and might be available for comparison in other study areas. For the FKM_A cluster, 0.66 and 0.13 of the variance was explained by the first and second axis, respectively, whereas for the FKM_B cluster, a greater amount of variance was explained by the second axis (0.55) relative to the first axis (0.15). For the FKM_C cluster, almost all of the variance was explained by the first axis (0.93). The high FKM_A and high FKM_C values were observed on opposite ends of the main gradient (Fig 3). This gradient was primarily from high median surface PHOS and high bottom PHOS range values, which are associated with high FKM_A values, toward high depth and high surface salinity values, which are associated with high FKM_C values. The FKM_C sites were positioned offshore at a distance from the effects of river inputs, whereas the FKM_A sites were those closest to the coastline and river inputs. The FKM_B sites were somewhat in the middle of this gradient, but only a rather small fraction of their variance was explained by this gradient. The high range of PHOS may have been related to more shallow areas, where occasional inputs of high river flow can affect the entire water column. Moreover, the bottom sediments in the shallow areas may be easily resuspended by vertical mixing and turbulence caused by waves and wind. The high concentrations of PHOS at depth, which is a signature of remineralization, have previously been described for the northern Adriatic Sea [58,87].

The second axis gradient mainly included the surface Vmean and Vmax, the median surface CPHL, the surface CPHL range, and the bottom TEMP range, with high FKM_B membership grades associated with low values of these variables and FKM_A membership grades associated with high values of these variables. The sites with high FKM_B memberships presented more of an offshore distribution relative to the FKM_A sites; thus, they were less influenced by riverine waters, which cause strong fluctuations in primary production because of seasonal fluctuations in river flow.

A portion of the variance could not be explained by our model, especially for the FKM_B membership grades (S1 Table). The distribution of high FKM_B values (Fig 1) revealed that several sites showing high FKM_A and FKM_B are located close to each other and many are also in the same cell within the 2.5 x 2.5 km grid on which the model was applied. Our resolution was constrained by the scarcity of available data; thus, it could not explain the observed differences between these sites. Moreover, the sites that were poorly fit by our model were found at a distance from each other in different parts of the study domain. This result suggests that certain local factors (e.g., fishing, sedimentation regimes, and endogenous factors such as autocorrelations caused by the clumping/dispersion of organisms) might have contributed to the observed variance in the outcrops.

Our results show that the surface and bottom dynamics are not always decoupled because of the limited depth of the water column in the study area. Thus, appropriate surface or bottom environmental descriptors can provide nearly equivalent explanations of the observed gradients in the outcrops (Table 4) notwithstanding possible causal relationships, which are not accounted for by the RDA. The depth range of the study sites was between 12.4 and 26 m, and even the deepest layers of the water column can be influenced by surface dynamics. Moreover, the height of the outcrops ranged from 0.5 to 4.5 m, and biotic data were collected on horizontal surfaces on top of the outcrops, which further reduced the possible effects of depth on the assemblages. In the study area, surface heat loss and wind-driven mixing in autumn and winter tend to homogenize the water column, but intense pulses of freshwater from rivers can induce relevant vertical stratification due to a layer of less saline water at surface. In spring and early summer, the vertical profile of temperature and salinity is strongly stratified with a noticeable thermocline; however, after strong wind events, the stratification can be broken and the mixed layer can reach the deepest parts of the water column. These wind events are less frequent in spring/summer than in autumn/winter.

**Table 4:** Portions of the variance explained by the three groups of variables (surface parsimonious model, bottom parsimonious model, and hydrodynamic parsimonious model) and depth. Only the effects of single groups, combinations of groups, and single groups conditioned to single groups and combinations of groups are shown. The (+) sign indicates that the variance is explained by that combination of variables. The (|) sign indicates that the variance explained by the group on the left is conditional on the variance explained by the group(s) on the right of the sign.

GROUP OF VARIABLES	adjusted R^2^	GROUP OF VARIABLES	adjusted R^2^
***Single groups***		***Conditional on 1 group***	
Surface	0.67	Surface|Bottom	0.09
Bottom	0.63	Surface|Hydro	0.48
Hydro	0.29	Surface|Depth	0.38
Depth	0.33	Bottom|Surface	0.05
***Combinations of 2 groups***		Bottom|Hydro	0.39
Surface+Bottom	0.72	Bottom|Depth	0.30
Surface+Hydro	0.78	Hydro|Surface	0.11
Surface+Depth	0.71	Hydro|Bottom	0.06
Bottom+Hydro	0.68	Hydro|Depth	0.21
Bottom+Depth	0.63	Depth|Surface	0.04
Hydro+Depth	0.54	Depth|Bottom	0.00
***Combinations of 3 groups***		Depth|Hydro	0.25
Surface+Bottom+Hydro	0.79	***Conditional on 2 groups***	
Surface+Bottom+Depth	0.73	Surface|Hydro+Depth	0.23
Surface+Hydro+Depth	0.77	Surface|Bottom+Depth	0.10
Bottom+Hydro+Depth	0.70	Surface|Bottom+Hydro	0.11
***All groups of variables***		Bottom|Hydro+Depth	0.16
All	0.79	Bottom|Surface+Depth	0.03
***Residuals (not explained)***		Bottom|Surface+Hydro	0.02
Residuals	0.21	Hydro|Surface+Depth	0.06
***Conditional on 3 groups***		Hydro|Bottom+Depth	0.07
Surface|Bottom+Hydro+Depth	0.09	Hydro|Surface+Bottom	0.08
Bottom|Surface+Hydro+Depth	0.02	Depth|Bottom+Hydro	0.01
Hydro|Surface+Bottom+Depth	0.05	Depth|Surface+Hydro	-0.01
Depth|Surface+Bottom+Hydro	0.00	Depth|Surface+Bottom	0.02

The high correlation of depth with selected surface and bottom environmental descriptors (Table 4) reveals that coastal-related processes, such as river inflows, play an important role in structuring the assemblages of the outcrops, whereas other processes, such as coastal pollution and recreational and commercial fishing, might also have an important role. In particular, the outcrops are threatened by mechanical damage related to trawling, heavy bottom gear disturbances, and anchoring. These practices are particularly destructive because of their direct effects, and they also increase the turbidity and sedimentation rates, which negatively affect the structure and composition of the assemblages. Encrusting calcareous macroalgae and *Polycitor adriaticus*, which are species that characterize Habitat C, are negatively correlated with the mud content of sediment [22,88]. In particular, *P*. *adriaticus* is found in undisturbed environments, and its populations are reduced or disappear at increased stress rates. Other tunicates, such as *Aplidium conicum*, which characterize Habitat B, are adversely affected by excessive sediment deposition, which causes burial and clogging of the siphons and the branchial wall [88]. Finally, the additive action of silting and high hydrodynamism has injurious consequences because the suspended inorganic particles have a mechanically abrasive effect on living organisms [89]. However, turfs are dominant in areas with increased sedimentation rates [23,79,90]. The abundance of encrusting sponges (i.e., *Dictyonella incisa*), which, together with turf algae, characterize Habitat A, increased with the mud and organic matter content of nearby sediment, whereas it decreased with increasing distance from the coast and increasing longitude and salinity [22].

Hydrodynamism appears to play an important role that is not shared among any of the other groups of variables (Table 4), and this result might be related to water renewal, advection in nutrient rich waters, variations in organism dispersal, and physical constraints on species that can cling onto the substrate. The sites with high FKM_A memberships are found close to the coast; thus, they are strongly affected by coastal currents that flow westward and south-westward in the study area and are seasonally enhanced by surface river inputs and meteorological conditions (easterly winds). These shallower areas display more energetic hydrodynamics throughout the year, whereas the areas characterized by high FKM_C membership appear to be occasionally affected by strong surface velocities that most likely do not affect the bottom assemblages because of the greater bottom depth. The FKM_B sites appear to be related to areas of weaker hydrodynamism; however, an inspection of the hydrodynamic subset RDA (S3 Fig) revealed that the FKM_B variance explained by the hydrodynamic variables was negligible. Thus, we can conclude that hydrodynamic variables do not play a role in differentiating FKM_B sites from the other two site clusters.

The onshore-offshore gradient is the most important gradient for explaining the variability of the assemblages growing over northern Adriatic biogenic outcrops because of the extent of coastal freshwater influence, which is the main driver of nutrient dynamics in the Northern Adriatic Sea, and the deepening of the water column in offshore sites, which lessens the sensitivity of the bottom population to certain surface dynamics (waves, surface) and is a proxy for the available light provided to the organisms growing on the outcrops. A less important gradient that is more difficult to explain according to the variables used in this study is confined to a coastal belt and differentiates two habitat types, FKM_B and FKM_A, with FKM_A experiencing greater exposure to environmental variability.

### Predictive model

**Fig 4:**
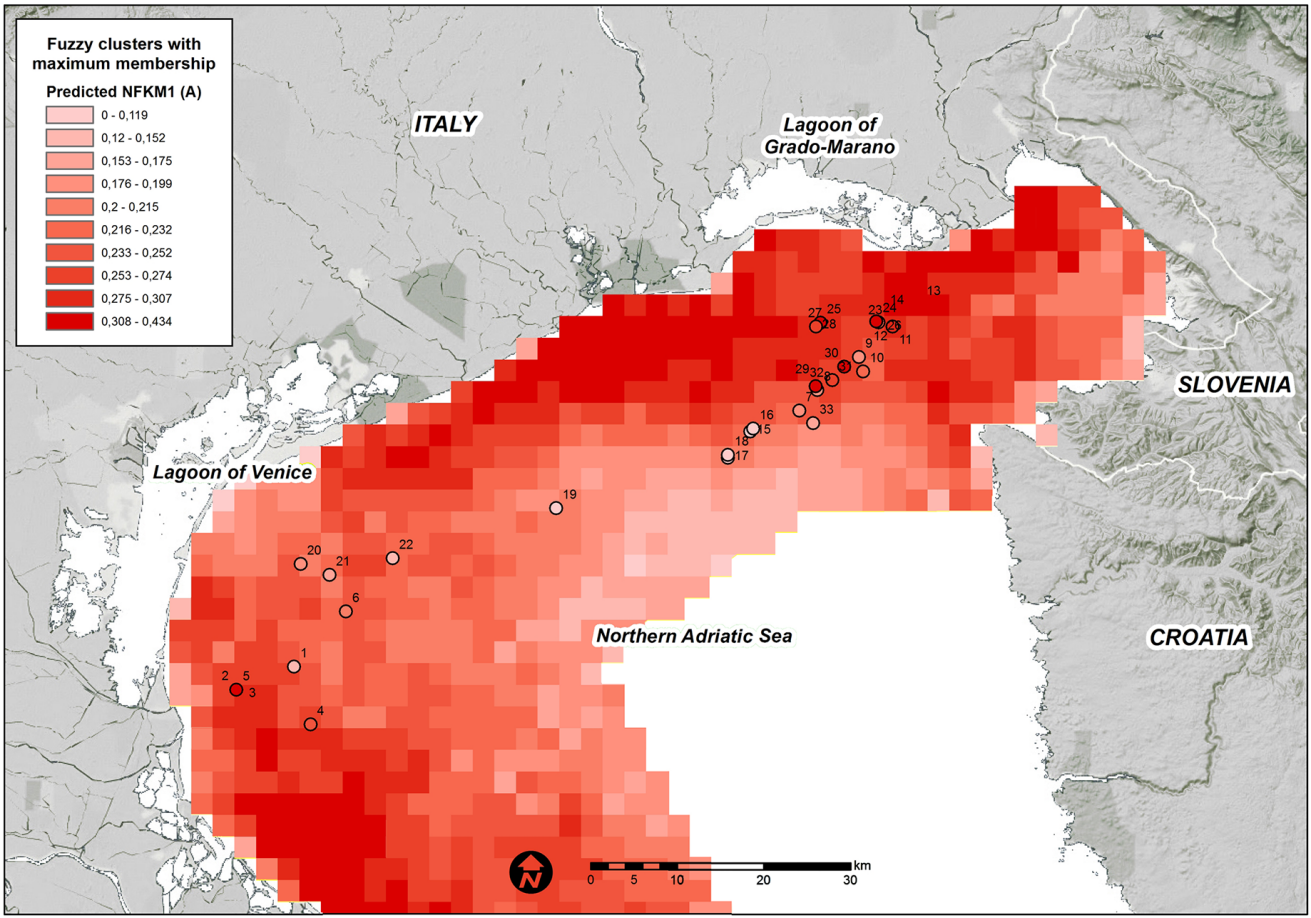
Predicted FKM_A memberships over the entire study area. Points show the sampling sites used in the present study. White = low membership and dark red = high membership.

**Fig 5:**
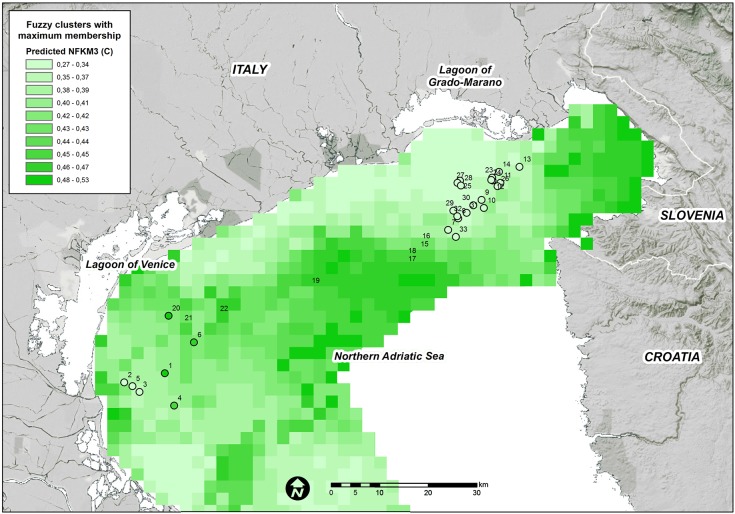
Predicted FKM_C memberships over the entire study area. Points show the sampling sites used in the present study. White = low membership and dark green = high membership.

**Fig 6:**
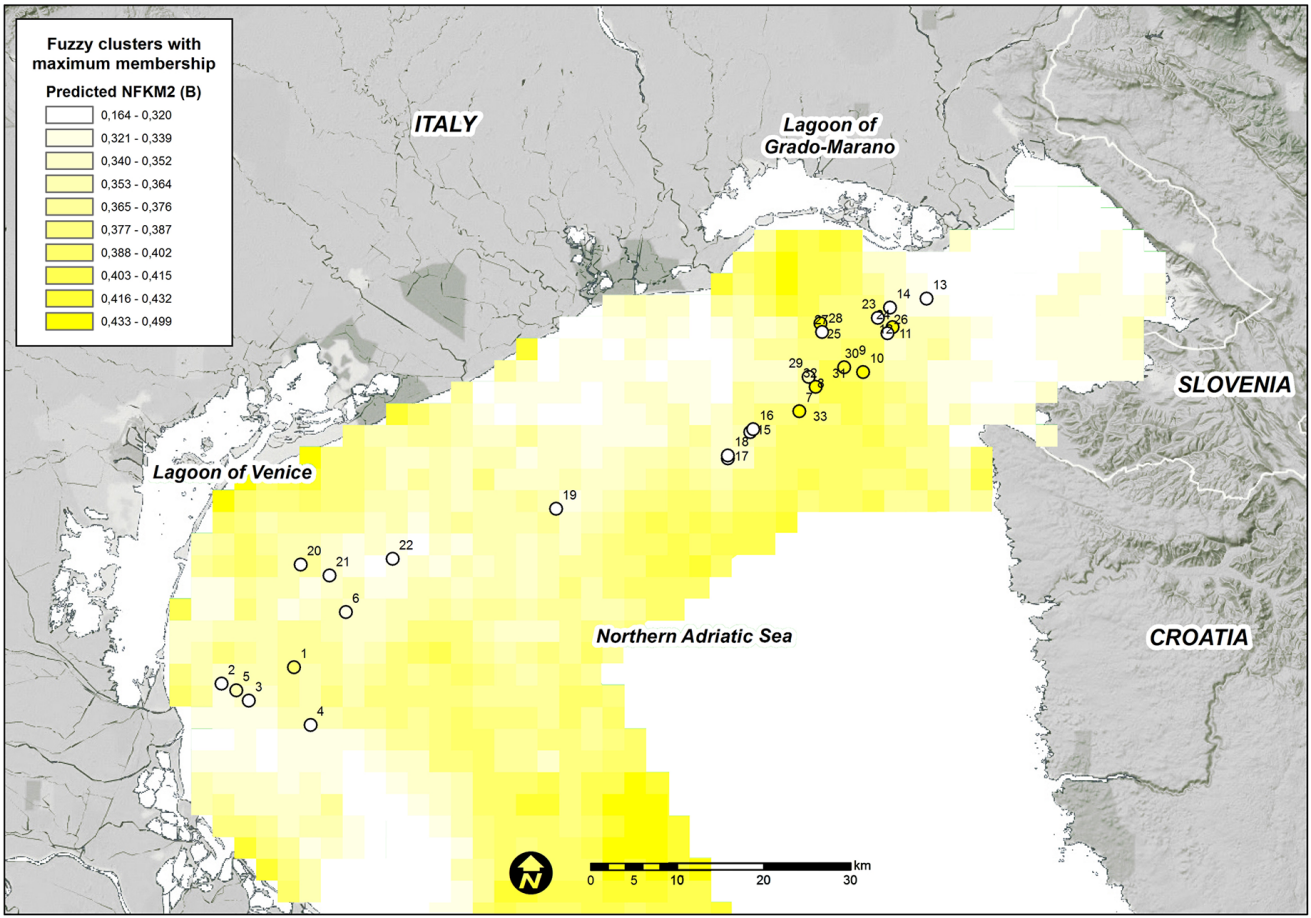
Predicted FKM_B memberships over the entire study area. Points show the sampling sites used in the present study. White = low membership and dark blue = high membership.

The final RDA model that was produced with 9 variables was used to predict the fuzzy membership grades of the three clusters over the entire study domain. The high predicted values for each fuzzy membership grade were generally consistent among the areas where they were observed (Figs 4, 5 and 6).

High FKM_A memberships are predicted along the coast, particularly in the north-western and south-western study area (Fig 4). The coastal belt in front of the Venice Lagoon and the Grado-Marano Lagoon are predicted to be less suitable for habitats in FKM_A. A few cells with high predicted FKM_A values are positioned in the Gulf of Trieste close to the mouth of the Isonzo River. In general, high FKM_A memberships appear to favor areas close to freshwater sources and areas at shallow bottom depths.

High FKM_C values are predicted offshore, at far distances from rivers and in deeper areas (Fig 5). In addition, the majority of the Gulf of Trieste as well as the coastal belt in front of the Venice Lagoon appear to be suitable for this cluster. The higher suitability of FKM_C compared with FKM_A in front of the Venice Lagoon might be a result of the buffer effect of the lagoon, which acts as a filter for high-nutrient loads transported to the lagoon from freshwater and from industrial and residential wastes [91,92].

FKM_B is predicted to occur close to the areas where this cluster has been observed, particularly in front of the Grado-Marano Lagoon (Fig 1). Nevertheless, the “intermediate” characteristics of the macrobenthic populations on the reefs of this cluster and its lower fit in the final RDA model compared with that of the other two clusters increase its likelihood in areas of the study domain where FKM_A or FKM_C (or both) are not predicted at high values (Fig 6). Areas of transition are a natural feature of the marine environment and may include a mixture of habitats and species.

The proposed model, that applies to the Italian side of the Northern Adriatic, and the actual occurrence of Habitat A, B, or C in the areas predicted by the model should be assessed with new samplings. Nevertheless, a major constraint that was not included in the model is the presence of a hard substrate. The presence or absence of a hard substrate is critical for the development of epibenthic communities; however, a complete cartography of substrate types in the study area is not available. Thus, our results might be helpful for defining areas worthy of exploration in further research projects.

Because mapping and comparing habitats across geographic regions is a key component of the classification process [15,17], the habitats derived from this study may be suitable for application in studies focused on other geographic areas. The Apulia continental shelf coralligenous outcrops fall into the “bank” category, which is similar to those in the northern Adriatic, and both contain the same features: isolated blocks randomly scattered on the soft bottom and clusters of blocks or ridges with several meters of lateral continuity [7,23]. These features could represent distinct phases of morphological development [7]. Outcrops with columnar shapes resembling small patch reefs also characterize the bottom off southeast Sicily [6]. If we consider the biotic component, the Apulian outcrops are colonized by coralline algae associated with organisms that also characterize the proposed habitats of the northern Adriatic; however, some of these outcrops show an additional “erect ramified” animal layer, thus representing a fourth complex habitat. The absence of larger bryozoans and gorgonians in the studied area is most likely related to the increased sediment resuspension, the reduced surface of colonization, and the high water turbidity.

Information on marine habitats must play a major role in ecosystem-based management promoted at national and international levels [93,94]. The three habitats proposed here are easy to identify in the field, and we have related these habitats to different environmental features (i.e., geography, nutrients, salinity, and temperature). We have also developed a predictive model based on environmental features, thus providing a large-scale probabilistic model of the presence of these different habitats in the northern Adriatic basin.

## Supporting Information

S1 Checklist Macroalgae reference checklist. (XLS)

S2 Checklist Epibenthic fauna reference checklist. (XLS)

S1 Fig Surface chemical-physical parsimonious RDA model. The adjusted R^2^ value for the entire model is 0.67, with 0.60 on the first axis and 0.07 on the second axis. Both axes are significant (p<0.01, 999 permutations).
(TIFF)

S2 Fig Bottom chemical-physical parsimonious RDA model. The adjusted R^2^ value for the entire model is 0.63, with 0.58 on the first axis and 0.03 on the second axis. Both axes are significant (p<0.05, 999 permutations).
(TIFF)

S3 Fig Hydrodynamic parsimonious RDA model. The first (and only significant) axis (p<0.001, 999 permutations) has an adjusted R^2^ = 0.26.
(TIFF)

S1 Table Fraction of the variance explained in the sampling sites. FKM is the fuzzy cluster with the highest membership at each site. RDA1 and RDA2 are the fractions of the variance explained by each axis, and TOT is the total fraction of the variance explained by the entire model.
(DOC)
