# Supplementary figures and images for: Calcareous Bio-Concretions in the Northern Adriatic Sea: Habitat Types, Environmental Factors that Influence Habitat Distributions, and Predictive Modeling
Source: PLoS One. 2015 Nov 11;10(11):e0140931. doi: 10.1371/journal.pone.0140931 (PMC4641629; doi:10.1371/journal.pone.0140931)

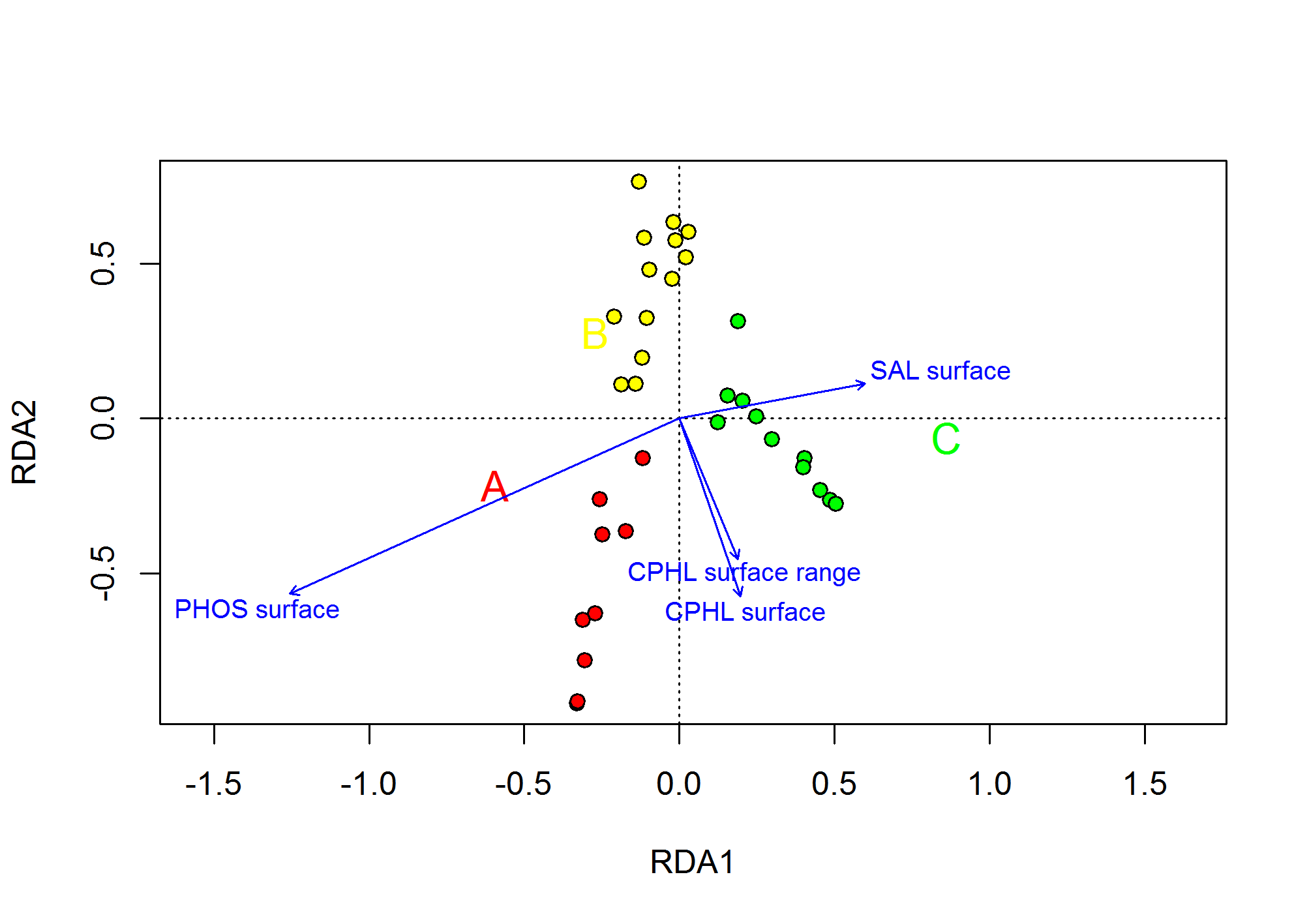

Supplement: S1 Fig — The adjusted R2 value for the entire model is 0.67, with 0.60 on the first axis and 0.07 on the second axis. Both axes are significant (p<0.01, 999 permutations). (TIFF) [file pone.0140931.s003.tiff]

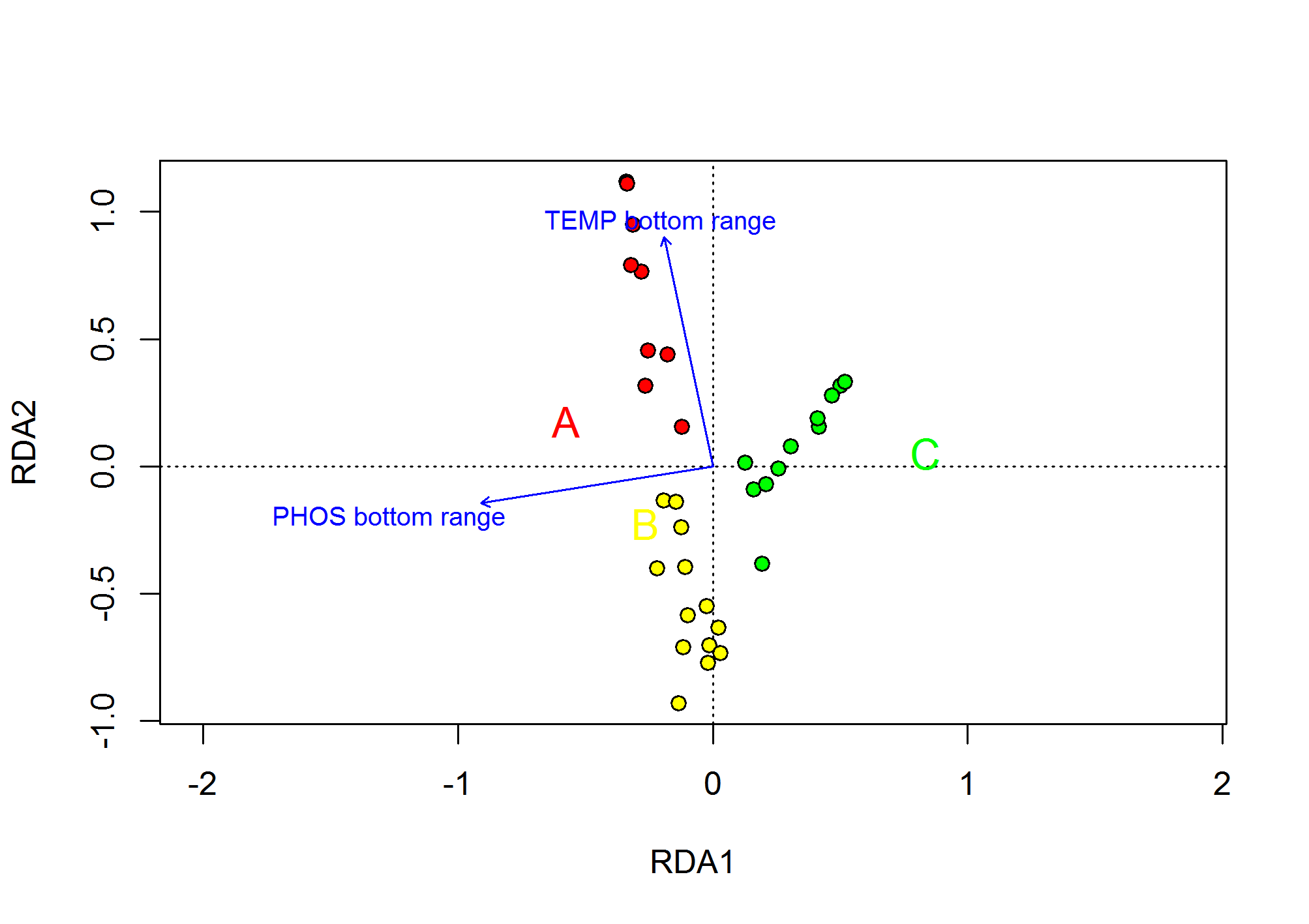

Supplement: S2 Fig — The adjusted R2 value for the entire model is 0.63, with 0.58 on the first axis and 0.03 on the second axis. Both axes are significant (p<0.05, 999 permutations). (TIFF) [file pone.0140931.s004.tiff]

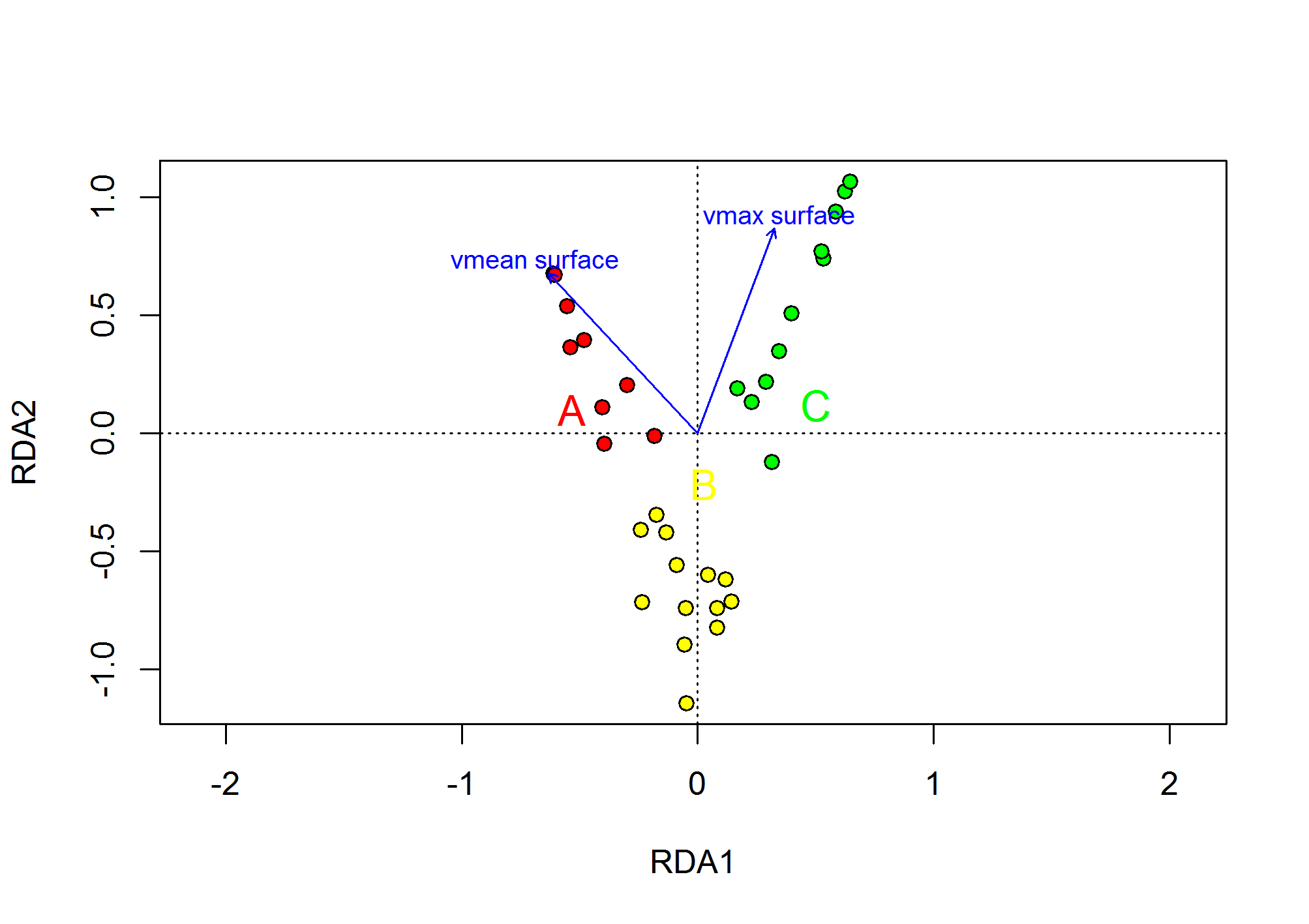

Supplement: S3 Fig — The first (and only significant) axis (p<0.001, 999 permutations) has an adjusted R2 = 0.26. (TIFF) [file pone.0140931.s005.tiff]
