# Supplementary material for: Calcareous Bio-Concretions in the Northern Adriatic Sea: Habitat Types, Environmental Factors that Influence Habitat Distributions, and Predictive Modeling
Source: PLoS One. 2015 Nov 11;10(11):e0140931. doi: 10.1371/journal.pone.0140931 (PMC4641629; doi:10.1371/journal.pone.0140931)
Supplement: S1 Table — FKM is the fuzzy cluster with the highest membership at each site. RDA1 and RDA2 are the fractions of the variance explained by each axis, and TOT is the total fraction of the variance explained by the entire model. (DOC) [file pone.0140931.s006.doc]

**S1 Table**

| **STATION** | **FKM** | **RDA1** | **RDA2** | **TOT** |
| --- | --- | --- | --- | --- |
| **ChioL1** | C | 0.54 | 0.23 | 0.77 |
| **ChioS1** | A | 0.26 | 0.06 | 0.32 |
| **ChioS3** | A | 0.76 | 0.22 | 0.98 |
| **ChioL2** | C | 0.51 | 0.20 | 0.71 |
| **ChioS2** | A | 0.59 | 0.22 | 0.81 |
| **ChioL3** | C | 0.79 | 0.07 | 0.86 |
| **TR12-Nicola** | B | 0.16 | 0.78 | 0.94 |
| **TR13** | B | 0.10 | 0.11 | 0.20 |
| **TR14-Misto** | B | 0.14 | 0.72 | 0.86 |
| **TR3-Spari** | B | 0.00 | 0.99 | 0.99 |
| **TR4** | B | 0.57 | 0.00 | 0.57 |
| **SanPietro** | B | 0.36 | 0.00 | 0.36 |
| **Menegh** | A | 0.70 | 0.29 | 0.99 |
| **Meneghel** | A | 0.72 | 0.26 | 0.98 |
| **Strucolo** | C | 0.88 | 0.06 | 0.94 |
| **Gubana** | C | 0.84 | 0.02 | 0.86 |
| **Colomba** | C | 0.97 | 0.02 | 0.99 |
| **Colomba2** | C | 0.94 | 0.02 | 0.95 |
| **Cerniotta** | C | 0.97 | 0.01 | 0.98 |
| **Lastre** | C | 0.87 | 0.04 | 0.91 |
| **Pivetta** | C | 0.94 | 0.02 | 0.96 |
| **Tartaruga** | C | 0.65 | 0.22 | 0.87 |
| **Amerigo** | A | 0.71 | 0.00 | 0.71 |
| **Corvine** | B | 0.74 | 0.00 | 0.74 |
| **NordAlti** | B | 0.95 | 0.00 | 0.96 |
| **Palo Largo** | A | 0.44 | 0.07 | 0.51 |
| **TR2-Pinnacoli** | B | 0.47 | 0.40 | 0.87 |
| **Salient** | A | 0.47 | 0.00 | 0.47 |
| **Saratoga** | A | 0.85 | 0.00 | 0.85 |
| **Dorsale** | B | 0.21 | 0.46 | 0.67 |
| **Aldebaran** | B | 0.14 | 0.82 | 0.96 |
| **La Longa** | B | 0.57 | 0.02 | 0.59 |
| **Bardelli** | B | 0.18 | 0.76 | 0.94 |
